# A brief overview of the size and composition of the myrtle rust genome and its taxonomic status

**DOI:** 10.1080/21501203.2014.919967

**Published:** 2014-05-29

**Authors:** Mui-Keng Tan, Damian Collins, Zhiliang Chen, Anna Englezou, Marc R. Wilkins

**Affiliations:** a NSW Department of Primary Industries, Elizabeth Macarthur Agricultural Institute, Private Bag 4008, Narellan, NSW 2567, Australia; b Systems Biology Initiative, School of Biotechnology and Biomolecular Sciences, The University of New South Wales, Kensington, NSW 2052, Australia; c Ramaciotti Centre for Genomics, The University of New South Wales, Kensington, NSW 2052, Australia

**Keywords:** *Puccinia*, phylogeny, transposons, DNA repeats, *Melampsora*, *Phakopsora*

## Abstract

Using *de novo* assembly of 46 million paired end sequence reads of length 250 bp for a myrtle rust isolate, we have estimated its genome size to be between 103 and 145 Mb and the number of proteins as >19,000. Annotation of the contigs found a very large percentage of proteins are associated with molecular functions of DNA binding or binding in biological processes for DNA integration and RNA-dependent DNA replication. A large proportion of these activities are attributed to the transposable elements (TEs). These elements are estimated to comprise 27% of the genome with 22% retrotransposons and 5% DNA transposons. The exon and intron boundaries of 46 genes occurring on contigs >20,000 bp have been determined. The number of introns range from 2 to 20 with a mean of 7. Phylogenetic analyses using partial *COXI, 18S rRNA* and *28S rRNA* genes have placed myrtle rust in the Pucciniaceae lineage on a separate taxonomic branch from the families of Pucciniaceae, Phragmidiaceae, Sphaerophragmiaceae, Phragmidiaceae, Uropyxidaceae, Chaconiaceae and Phakopsoraceae. Further work is thus required to determine the family placement of myrtle rust in the Pucciniaceae of Pucciniales.

## Introduction

Myrtle rust (*Puccinia psidii* sensu lato) was reported for the first time in Australia in April 2010 from *Agonis flexuosa, Callistemon viminalis* and *Syncarpia glomulifera* (Carnegie et al. 2010). These rusts are serious pathogens which infect plants in the family Myrtaceae, including Australian natives like bottle brush (*Callistemon* spp.), tea tree (*Melaleuca* spp.) and eucalyptus (*Eucalyptus* spp.). Since its first report, the disease is currently found to be widely distributed along the entire east coast of New South Wales and parts of Queensland and Victoria.

Myrtle rust produces masses of powdery bright yellow or orange-yellow spores on infected plant parts. It infects susceptible plants producing spore-filled lesions on young actively growing leaves, shoots, flower buds and fruits (Carnegie et al. 2010). Leaves may become buckled or twisted and may die as a result of infection. Sometimes these infected spots are surrounded by a purple ring. Older lesions may contain dark brown spores. Infection on highly susceptible plants may result in plant death.

Myrtle rust was initially named *Uredo rangelii* (Carnegie et al. 2010) because it is morphologically distinct from *Puccinia psidii* (Winter 1884) which was the name first used for guava rust. The teliospores from myrtle rust which were found later, matched those of *P. psidii* sensu stricto (Simpson et al. 2006), and this led to its name being revised to *P. psidii* sensu lato (Carnegie and Lidbetter 2012). The pathogen is referred in this paper as myrtle rust to distinguish it from guava rust.

*Puccinia psidii* s.l. had been reported in many countries and has a wide host range across genera (Glen et al. 2007; Roux et al. 2013). A strain native to South America is very damaging to *Eucalyptus* plantations in Brazil (Junghans et al. 2003) and hence is commonly referred as eucalyptus rust. Some of the *Eucalyptus* species introduced for commercial purposes originated from Australia. Eucalyptus rust has since been reported in majority of countries in South and Central America, Florida, California and Hawaii in the United States and considered a serious threat to eucalyptus plantations worldwide (Coutinho et al. 1998; Grgurinovic et al. 2006).

Myrtle rust has been documented on 107 host species in 30 genera from data collected during the 2010 surveys in the Australian state of New South Wales under the state emergency response program (Carnegie and Lidbetter 2012). Host range studies performed using artificial inoculation experiments (Carnegie and Lidbetter 2012) showed that several species of Australian *Eucalyptus* are susceptible to myrtle rast. There is to date no report of a natural infection on *Eucalyptus* in Australia.

The threats myrtle rust poses to the Australian native flora and the forestry industry worldwide make it crucial to have an in-depth genetic understanding of the fungus. There is to date very limited genetic data available for eucalyptus/guava rusts, and they were thus unable to shed any light on the myrtle rust fungus. The taxonomic status of *P. psidii* has been reported in a study using the ITS and 5.8S gene involving a very limited number of *Puccinia* taxa (Roux et al. 2013).

The objective of this study was to obtain a brief overview of the size and composition of the myrtle rust genome using a cost-effective, high throughput sequencing technology and the determination of its taxonomic status. This will provide some genetic understanding of the pathogen for the development and implementation of long-term management. The genetic data from genome sequencing has enabled a more rigorous taxonomic study involving more genetic regions and a larger number of rust taxa from the Pucciniales.

## Materials and methods

### DNA extraction and genome sequencing

Multiplication of a single urediospore of the myrtle rust pathogen, PBI accession no, 115012-Mr was performed by inoculating it on the host, *Syzygium jambos* (rose apple) at the Cereal Rust Research Unit, Plant Breeding Institute, University of Sydney. About 0.1 g of spores was ground in extraction buffer (50 mM *tris*-HCl [pH 8.0], 0.7 M NaCl, 10 mM EDTA, 1% [wt/vol] cetyl trimethy-lammonium bromide [CTAB; Sigma H-5882], and 1% [vol/vol] 2-mercaptoethanol) for DNA extraction. High-quality, un-degraded DNA was extracted as outlined by Tan and Niessen (2003).

A shotgun library of sequences of about 650 bp was prepared using the TruSeq DNA Sample Preparation kit (http://www.illumina.com/). The library of random DNA fragments from the entire genome was sequenced on the MiSeq Sequencer according to the manufacturer's instructions (http://www.illumina.com/systems/miseq.ilmn) at the Ramaciotti Centre for Genomics, University of NSW. Two separate sequencing runs were performed from the library.

### Sequence processing and assembly

The combined sequences from both runs were trimmed to remove low-quality sequences using the ‘trim’ tool in the CLC Genomics Workbench 6 (www.clcbio.com). A limit value of 0.05 was used for the quality trimming with a maximum number of two ambiguous nucleotides at the sequence ends allowed.

A number of assembly programs were investigated and compared, including open source software, Readjoiner (Gonnella and Kurtz 2012), String Graph Assembler (SGA) (Simpson and Durbin 2012), ABySS (Simpson et al. 2009), SOAP denovo (Luo et al. 2012) and the commercial software, CLC-Bio Genomics Workbench. Assemblies were reiterated with different overlap length for Readjoiner and SGA and different *k*-mer values for ABySS, SOAPdenovo and CLC-Bio Genomics Workbench to obtain the highest N50 value and the longest contigs. The following parameters were applied for the CLC-Bio Genomics Workbench: mismatch, insert and deletion cost = 3; length fraction = 0.5; similarity fraction = 0.75; minimum contig length = 1000; Bubble size = 250; mapping mode = map reads back to contigs.

### Contig analysis

*De novo* assembly data used for analysis was the set of contigs with length, *l* ≥ 3000 bp generated by the assembly program, CLC-Genomics Workbench which gave the highest N50 value (Table 1). Contigs in the range 3000–8000 bp were blasted, mapped and annotated using the Blast2GO software (http://www.blast2go.com/b2ghome).

**Table 1.** A comparison of genome assembly results for myrtle rust using different *de novo* assemblers.

*De novo* assembler		Total # of contigs	N50^3^ (bp)	Max Contig (bp)	Total length (bp)
**Readjoiner** (*ol* = 70)	all	4,963,090	439	3099	2,147,976,637
	^2^*l* ≥ 750	116,914	859		103,828,908
**SGA** (^1^*ol* = 85)	all	1,321,742	493	8631	573,387,137
	*l* ≥ 750	135,453	976		137,671,501
**AbySS** (*k* = 45)	all	759,095	558	25,668	362,000,000
	*l* ≥ 750	99,806	1522		145,365,606
**SOAPdenovo** (*k* = 127)	all	766,280	567	6094	387,909,377
	*l* ≥ 750	109,692	949		108,060,557
**CLC-Bio** (*k* = 40)	all	148,139	3165	47,187	387,958,276
	*l* ≥ 750	148,138	3165		387,956,878
	*l* ≥ 3000	37,684	5535		203,507,520
	*l* ≥ 4000	23,106	7929		153,506,636

Notes: ^1^overlap length;

2 contig length (bp);

3 An N50 contig size of *N* means that 50% of the assembled bases are contained in contigs of length *N* or larger. N50 sizes are often used as a measure of assembly quality because they capture how much of the genome is covered by relatively large contigs.

Contigs longer than 8000 bp were blasted using the Blastx program on the NCBI site (http://blast.ncbi.nlm.nih.gov/Blast.chi). The results were imported into a Blast2GO file for mapping and annotation.

All blastx analyses were run against the fungal set of non-redundant protein sequences, with a word size of 3, expectation value of 10.0, and the number of blast hits archived was limited to 5. The scoring parameters were BLOSUM62, Existence: 11, Extension: 1, with conditional compositional score matrix adjustment.

The blast results (blast result accessions) were mapped using Blast2GO to retrieve gene ontology (GO) terms associated with the hits. The mapping step was followed by GO annotation using the default parameters in the Blast2GO program. The annotated sequences were then analysed using the data mining tool in Blast2GO with respect to the distribution of the annotated sequences in the cellular component, molecular function and biological process of the genome (Götz et al. 2008).

### Determination of proportions of repeats and their coverage

Text strings for repeated sequences were used to match against the ‘sequence description’ of the Blast2GO output. They were retrotransposable elements (pol, gag, rve, integrase (IN), polyprotein, retrotransposable, retrotransposon, tick, reverse transcriptase (RT), RNAse H); copia polyprotein (Tyl, copia); gypsy retrotransposon (Ty3, gypsy, nucleocapsid); DNA transposons (Tcl, hAT, DDE, mutator, transposase); hAT; mutator; Tcl. These text strings were used to calculate the number of contigs with these repeat elements in the contig set, *l* ≥ 3000 in the Blast2GO document file.

The mapping results of the Blast2GO output were exported in a tab-delimited text file and merged with the coverage report of the CLC-Bio contig set *l* ≥ 3000 bp to calculate the mean, median, minimum and maximum coverage of the contigs with the various classes and families of transposable elements (TEs). A distribution of contig coverages for all contigs was also plotted for reference.

### Fine manual annotation of protein-coding genes

Blast results with significant homology (*E*-value < −50) with closely related gene orthologs from other fungal organisms, e.g., *P. graminis* were used as starting points to perform fine annotation of genes on the set of contigs with length > 20,000 bp. Each of the query contig from myrtle rust with a highly significant blastx result (*E*-value < −50) for a protein gene will have multiple segments of the contig matched to segments from the five archived orthologous gene sequences with different homology values (*E*-value). The nucleotide interval of the query contig that had the highest homology (lowest *E*-value < −50) was used as an anchor point to determine the putative boundary of that exon.

Using the blast result as a guide, a nucleotide interval downstream or upstream of the putative boundary of that exon was then selected. This selection was translated in three different frames and aligned with corresponding protein segments of the gene orthologs. The frame that gave the highest homology was used to determine the boundaries of the intron and the adjacent exon. The process was repeated until all exon sequences of the gene were determined.

### Phylogenetic analysis

The *COX1* (KF477285), the nuclear *28S rRNA* (KF792096) and the *18S rRNA* (KF792096) genes of myrtle rust were used to determine its phylogenetic position in the rust complex. Phylogenetic analysis of DNA sequences was performed using the criteria of maximum parsimony, distance and likelihood in the programs PAUP* Version 4b10 (http://paup.csit.fsu.edu/) and MEGA6 (www.megasoftware.net). Bootstrap with 1000 replicates was used to assess the relative strength of branches for maximum parsimony and distance criteria and 100 replicates for maximum likelihood method.

## Results

### Sequence assembly and estimation of genome size

Two separate next-generation sequencing (NGS) runs performed on a MiSeq Sequencer generated 18 million and 28 million paired-end reads of 250 bp per read. The combined sequences from both runs were trimmed to remove low-quality reads, which reduced the average length from 250 bp to 242 and 234 bp for Run 1 and Run 2, respectively. A total of 24,953,737 of the 46,322,773 reads were trimmed with 6187 reads discarded. The ambiguity trim removed 88,821 reads (Table S1 in ‘Supplementary Material').

High-quality trimmed sequences were assembled using a number of separate DNA assemblers, and the outputs were then compared. This was necessary as it is impossible to predict which program will give the best assembly for any particular genome. Two string graph-based open-source software, Readjoiner (Gonnella and Kurtz 2012) and SGA (Simpson and Durbin 2012) are fast and memory efficient and were first used. Using Readjoiner (Table S2) with different overlap lengths gave N50 values in the range 432–440 bp. The SGA gave a very similar N50 of 493 but with a much longer maximum contig length of 8631 bp (Table 1).

DNA assemblers based on de Bruijn graph (Pevzner et al. 2001) approach the computational task by breaking reads into smaller sub-sequences of DNA, called *k*-mers, where the *k* parameter describes the length in bases of these sub-sequences (Miller et al. 2010). The *k*-mer length used in the ABySS software ranged from 45 to 89, whereas the length used in the CLC-Bio Genomics Workbench ranged from 32 to 64 (the longest length allowed). The ABySS software gave N50 values in the range 515–558 bp and maximum contig sizes from 20,332 to 35,730 bp (Table S3). The maximum contig size was not directly proportional to the N50 values (Table S3). The SOAPdenovo (http://soap.genomics.org.cn/) assembly program gave a N50 = 976 bp and a max contig length = 6094 bp with a *k*-mer = 127 (Table 1). These two ‘de Bruijn graph’ assemblers performed slightly better than the two ‘string graph-based’ assemblers on the N50 measure.

The CLC-Genomics workbench gave the highest N50 value of 3165 bp and a maximum contig size of 47,817 bp with *k*-mer value of 40 (Table 1, Table S4). Thus, a comparison of the outputs from the different assembly programs (Table 1) indicated the best output as one obtained using *k*-mer = 40 on the CLC-Bio Genomics Workbench.

The sets of contigs assembled using the various softwares (Table 1) were used to estimate the genome size. For assemblies with N50 values around 500 bp, the sets of contigs with threshold length of at least 750 bp were summed to give an estimate of genome size. Hence the genome size was estimated to range from 103 Mb (Readjoiner) to 145 Mb (ABySS). The sum of contig lengths ≥750 for the assembly from CLC-Bio Genomics Workbench was a much larger value as expected because the N50 value was much higher (Table 1). For the N50 of 3165 bp in the CLC-Bio contig set, the genome size was estimated to be in the range between the sum of contigs for l ≥ 3000 bp and l ≥ 4000 bp, which gave the estimate between 153 and 203 Mb (Table 1).

### Blast analysis

The set of contigs, with length, *l* ≥ 3000 bp obtained from the CLC-Bio Genomics Workbench (Table 1) was matched against non-redundant protein sequences (nr database at NCBI) with Blastx to identify protein coding regions. The highest number of matches was obtained with the stem rust species, *P. graminis*, and the second largest number of matches was with another rust species, *Melampsora larici-populina.* The protein composition of myrtle rust genome was estimated from the contig set, *l* ≥ 3000 bp and comprises −10,105 hypothetical proteins, 6800 mapped proteins and 2907 annotated proteins (Table 2), giving the estimated number of proteins as 19,812 (Table 2). The CLC-Bio contig sets, *l* ≥ 3000 bp and *l* ≥ 4000 bp (Table 1) have 2907 and 2032 proteins annotated, respectively, using the default parameters in Blast2GO (Table 2).

**Table 2.** Protein composition in the myrtle rust genome.

	Contig set *l* ≥ 3000 bp		Contig set *l* ≥ 4000 bp	
Proteins	# of contigs	%	# of contigs	%
Total # of contigs	37,605		23,106	
No blastx hits	17,714	47.0	9150	39.6
Mapped proteins	6800	18.1	4932	21.3
Blast2GO annotated proteins	2907	7.7	2032	8.8
Mitochondrial contigs	79	0.21	65	0.28
Hypothetical proteins	10,105	26.9	6927	30
-[Uncharacterized protein	[21	[0.1	[16	[0.1
hypothetical protein ^1^PGTG_XXXXX	8803	23.4	5994	25.9
hypothetical protein	704	1.9	496	2.1
^2^MELLADRAFT_XXXXX	19	0.1	17	0.1
hypothetical protein	30	0.1	20	0.1
^3^TREMEDRAFT XXXXX	8]	0]	7]	0]
hypothetical protein ^4^MPER_XXXXX Others]				

Notes: [] indicates the breakdown of hypothetical protein.

1 PGTG_XXXXX: hypothetical protein [*Puccinia graminis f. sp. tritici* CRL 75-36-700-3].

2 MELLADRAFT_XXXXX: hypothetical protein [*Melampsora larici-populina* 98AG31].

3 TREMEDRAFT_XXXXX: hypothetical protein [*Tremella mesenterica* DSM 1558].

4 MPER_XXXXX: hypothetical protein [*Moniliophthora perniciosa* FA553].

**Figure 1.**
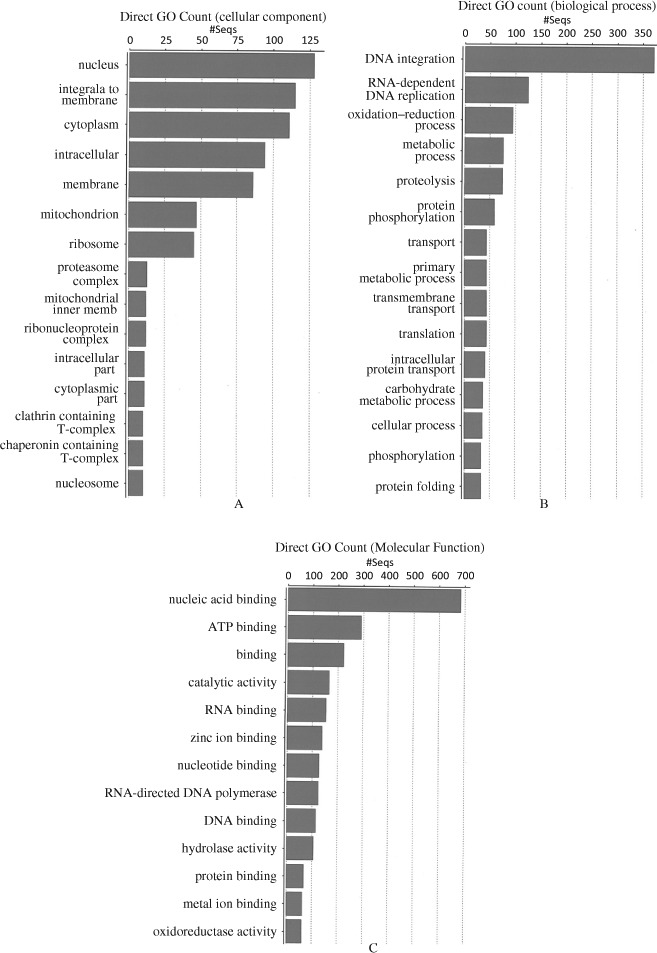
Direct counts of sub-lists of GO terms for cellular components (A), biological process (B) and molecular function (C) in the contig set, *l* ≥ 3000 of the myrtle rust genome.

GO counts in the cellular component include large numbers for proteins in the nucleus, cytoplasm, membrane and mitochondrion (Figure 1). Proteins for DNA integration and RNA-direndent DNA replication (Figure 1) were strongly represented in the group of GO terms in biological process. Proteins in these categories comprised largely those from TEs. The GO terms in molecular function (Figure 1) are very strongly associated with binding, with a large proportion involved in nucleic acid binding (Figure 1). RNA binding and RNA-directed DNA polymerase activities are also significant indicating the presence of retrotransposons. The distributions of all the annotated sequences in the cellular component, biological process and molecular function of the genome are available on request.

### Transposable elements

TEs are short, mobile, conserved segments of DNA that can replicate and randomly insert copies within genomes. Eukaryotic TEs are divided into two classes, Class I (retrotransposons) and Class II (DNA transposons). The proportion of TEs estimated for the myrtle rust genome was about 27%, with the Class I LTR retrotransposons present in a much higher ratio of about 22% (Table 3). The DNA transposons (Class II elements) are grouped into super-families based on sequence similarity of the element-encoded transposase. The proportions of three superfamilies have been estimated (Table 3) with the mutator family accounting for −19% of the DNA transposons (Table 3).

**Table 3.** Percentages of different classes of TEs in contigs, *l* ≥ 3000 bp and *l* ≥ 4000 bp from CLC-Bio Genomics Workbench.

	*l* ≥ 3000 bp				*l* ≥ 4000 Dp				
							Total length	
TEs	# contigs	%	Total length (nucleotide)	%	# contigs	%	Total length (nucleotide)	%
Class I:								
LTR retro-transposons	7995	21.2	43,373,367	21.4	4930	21.4	32,851,702	21.6
Class II:								
DNA transposons	1626	4.3	10,648,124	5.3	1153	5.1	9,019,956	5.8
^1^*[*	[	[	[	[	[	[	[	[
-mutator	311	19.1	2,040,658	19.1	223	19.3	1,739,036	19.3
-hAT	56	3.4	417,836	3.9	42	3.6	369,208	4.1
-Tel	29	1.8	161,062	1.5	20	1.7	129,046	1.4
]	]	]	]	]	]	]	]	]

Note: ^1^[] indicates the breakdown of DNA transposons.

**Figure 2.**
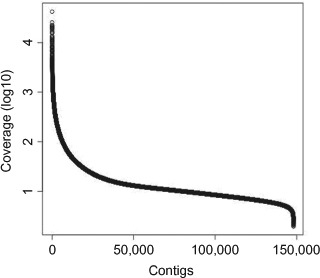
Distribution of coverage (log_10_ scale) of all contigs from the CLC-Bio Genomics Workbench assembled data.

A plot of the distribution of coverage (logio scale) of all CLC-Bio contigs gave a mean coverage of 10.54 (Figure 2), which is similar to that obtained for *P. striiformis* (Cantu et al. 2011). The contigs for retrotransposable elements and DNA transposons have higher mean coverages of >20 with maximum coverages of more than 10,000 (Table 4).

**Table 4.** Distribution of mean, median, minimum and maximum coverages of TEs and hypothetical protein in the contig set, *l* ≥ 3000 bp and *l* ≥ 4000 bp.

	Contig set with *l* ≥ 3000 bp				Contig set with *l* ≥ 4000 bp			
Proteins	Mean	Median	Min	Max	Mean	Median	Min	Max
Retrotransposable elements	21.5	11.55	4.22	11,286.87	21.4	11.59	4.23	11,286.87
Copia polyprotein	16	11.2	4.91	796.6	14.5	11.43	5.02	412.22
Gypsy retrotransposon	22.5	12.03	4.62	944.55	18.3	12.00	4.83	538.69
DNA transposons	26.4	11.315	4.61	11,188.22	13.8	11.6	5.23	462.26
HAT	12.7	11.57	6.7	40.34	12.2	11.84	6.7	24.57
DDE	12.4	10.61	5.47	113.58	13.1	11.34	5.8	113.58
Mutator	16.3	11.32	4.93	583.75	13.3	11.57	5.54	100.35
Tcl	11.6	11.33	5.76	18.13	11.5	11.22	5.76	18.13
RNase H	18.7	11.5	4.44	896.15	15.4	11.82	5.99	255.31
Hypothetical protein	16.7	10.91	3.85	5704.75	16	11.05	4.33	5704.75

### Fine annotation of myrtle rust genes

The boundaries of exons and introns were determined for 46 protein genes on contigs with length >20,000 nt. The number of exons ranges from 2 to 20 with a mean of 7 (Table 5). These contig sequences with their protein gene annotations have been submitted to GenBank (Table 5). The ribosomal unit comprising *28S rRNA* gene, ITS1, *5.8S* gene, ITS2 and the *18S rRNA* gene were annotated on a contig of length 6326 bp (KF792096) which has an average coverage of 5705.

**Table 5.** Annotated genes of the myrtle rust pathogen with their corresponding GenBank accession numbers.

Gene	# exons	Contig length	GenBank accession #
AarF; Pkc_like	5	22,551	KF431993
AdoMet Mtases	6	22,485	KF431980
ATP12	7	22,485	KF431980
ATP_sub_h	2	20,659	KF431974
C2_RasGAP	3	33,008	KF431975
Clathrin-associated protein	9	23,635	KF431979
Cpn60_TCPl	8	20,034	KF431976
DNA repair protein (radl)	14	32,354	KF431977
DnaJ	7	21,047	KF431981
DSPc	6	20,930	KF431982
Eukaryotic translation initiation factor 2 subunit	7	26,736	KF431983
Farnesyl-diphosphate farnesyltransferase	8	24,879	KF431984
FAT; TRRAP; PI3Kc	14	31,264	KF431988
GITSHD	10	21,927	KF431985
Glyco hydro 2 C	12	28,805	KF431986
Glyco transf 25	2	22,428	KF431989
Glycoside hydrolase family 92 protein	20	20,606	KF431987
Heterokaryon incompatibility protein Het-C	17	26,736	KF431990
IbpA ACD LpsHSP like	3	33,590	KF431991
MBOAT2	3	21,660	KF431992
nadF	5	21,393	KF431994
Patatins and Phospholipases	6	31,522	KF431995
Pectate lyase 3	2	22,483	KF431996
Peptidase C14 (Caspase domain; pfam656)	11	24,405	KF431997
Peptidase_M 14NE-CP-C_like	6	29,845	KF431998
peptidase M17	9	25,580	KF431999
Peptidylprolyl isomerase	4	24,879	KF432000
Phox homology (PX) domain protein (COG5391)	8	20,306	KF432001
rab family protein	4	22,719	KF432002
Ras-like protein Rab7	6	30,285	KF432003
Ribosomal PO like	5	37,821	KF432004
RINT-1 TIP-1 family; pfam4,437	9	20,112	KF432005
SCAMP family; pfam4,144	5	24,168	KF432006
SecE	4	20,357	KF432007
Sen 15	4	22,496	KF432008
SF3bl HSH155	13	21,811	KF432009
Sfil	11	23,803	KF432010
Small nuclear ribonucleoprotein D3	4	24,696	KF432011
SPX_CitT_SLC13 permease	5	20,601	KF432012
Sun_AdoMet_MTases	10	20,962	KF432013
TFIIE beta winged helix	7	23,401	KF432014
Ubiquitin thiolesterase	9	20,356	KF432015
Ubox_RING_cyclophilin_RING	11	29,845	KF432016
Uncharacterized conserved protein COG0397	5	20,101	KF432017
WD40 Peptidase CI9 UCH 1 PAN2 exo	10	22,935	KF432018
COXI gene	11	38,639	KF431978
28S-ITS-18S rRNA unit	–	6326	KF792096

### Phylogenetic analysis

The 5′end of the *28S rRNA* gene comprising 542 nucleotides were aligned with homeologous sequences from 50 taxa (Maier et al. 2003) for phylogenetic analysis with *Herpobasidium filicinum* (AF426193) defined as an out-group. The *18S rRNA* gene comprising 1005 and 434 nucleotides from the 5′end and 3′end, respectively, of the gene were aligned with 23 taxa (Wingfield et al. 2004) for phylogenetic analysis with *Atelocauda digitata* (AY125400) defined as an outgroup. Neighbour-joining analysis using various distance models including Jukes-Cantor, *p*-distance and Kimura 2-parameter; and analysis using heuristic search with maximum parsimony or maximum likelihood methods in the programs PAUP4 and MEGA6 gave congruent outcomes in the taxonomic placement of myrtle rust.

**Figure 3.**
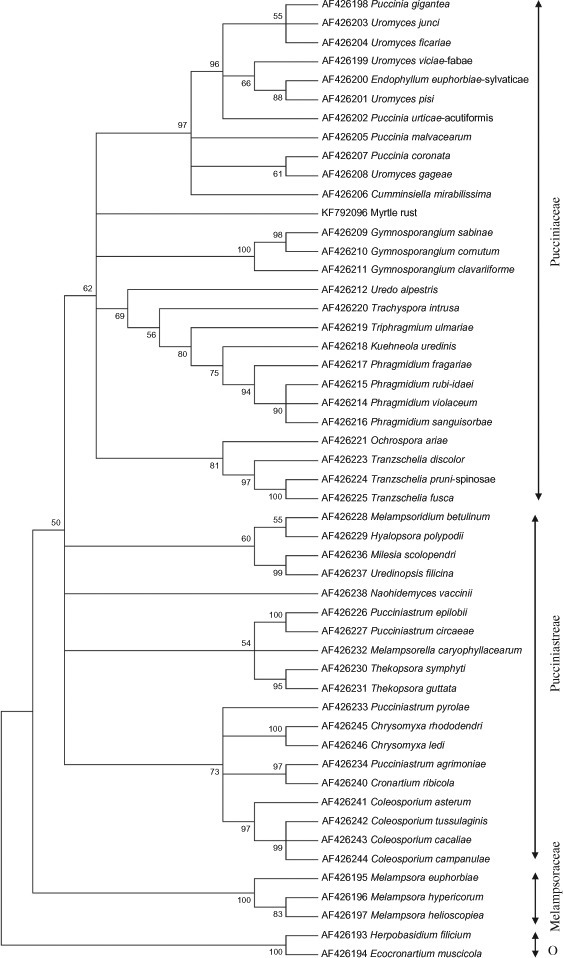
A consensus tree generated using the neighbour joining method on the Jukes-Cantor model with 1000 bootstrap replicates in the program MEGA 6.0 for the *28S rRNA* gene sequence (5′ end; 543 characters) from 51 different rusts including myrtle rust. Only bootstrap values greater than or equal 50% of 1000 replicates are shown. The GenBank accession numbers of the DNA sequences are indicated with the species name. Outgroups (O) are *Herpobasidium filicium* and *Ecocronartium muscicola.*

The analysis clustered myrtle rust with the Pucciniaceae clade on a branch separate from the representatives of the families of Pucciniaceae (Gymnosporangium, Puccinia/ Uromyces); Phragmidiaceae, Sphaerophragmiaceae, Phragmidiaceae, Uropyxidaceae, Chaconiaceae and Phakopsoraceae (Figure 3 and Figure S1).

The complete *COXI* gene of myrtle rust (GenBank KF431,978) annotated in this work has 11 exons, which with introns span a total length of 18,670 nucleotides. The gene's exons are a total of 1620 nucleotides, coding 540 amino acids. Due to the large number of introns in the *COXI* gene of basidiomycetes, complete *COXI* gene sequences are limited to date to five species.

A short segment (81 amino acids) coding the 5′ end of the *COXI* gene was available in GenBank for many *Puccinia* and *Melampsora* species. These were aligned with orthologous regions of the *COXI* gene from myrtle rust (KF431978) and two *Phakopsora* species (YP 3795694, AE098525; YP 3795384) for analysis. Alignment was also made for the corresponding coding DNA sequences.

Analysis of the DNA coding sequences of the partial *COXI* gene fragment with the fungus, *Ustilago maydis* as an outgroup, also suggested myrtle rust to belong to the Pucciniaceae clade of the Pucciniales and on a separate branch from the one for the cereal rust fungi and the *Phakopsora* species (Figure S2).

## Discussion

This work generated 46 million paired end reads of read length 250 nucleotides on the Illumina MiSeq platform. There is no reference genome for the myrtle rust. Contigs were assembled *de novo.*

Assembly software based on overlap graphs, Readjoiner and SGA, did not perform as well as software based on the de Bruijn graph. Of the assemblers based on the de Bruijn graph, the CLC-Genomics Workbench gave the highest N50 value and the longest ‘maximum contig length’ (Table 1).

The genome size of the myrtle rust pathogen was estimated to be between 103 and 145 Mb (Table 1). This size is relatively large for fungal genomes (Baker et al. 2008), but comparable to the size of rust genomes reported including *P. triticina* (100–120 Mb) and *P. Striiformis* (110 Mb) (www.broadinstitute.org) and *Melampsora larici-populina* (101 Mb; Duplessis et al. 2011).

The number of proteins in the genome may be as many as 19,000 (Table 2). This number is in the range of the number of proteins estimated for cereal rust genomes which range from 14,878 *P.* *triticina* to 19,542 *P.* *striiformis* (http://www.broadinstitute.org/annotation/) and 16,399 and 17,773 for *M*. *larici-populina* and *P. graminis* f. sp. *tritici*, respectively (Duplessis et al. 2011).

This work has shown that the myrtle rust genome has a large fraction of repeated elements of which a significant percentage is TE. The proportion of TEs was estimated to be about 27% (Table 3). This is in agreement with recent findings of whole genome sequencing of rust genomes including *P. striiformis* f. sp. *tritici* (Cantu et al. 2011) and the basidiomycete *Laccaría bicolor* (Labbe et al. 2012), which reported a percentage of 17.8% and 24%, respectively. Other rust species have been reported to have much higher percentage of TEs. The rust species, *Melampsora larici-populina* and *P. graminis* f. sp. *tritici* were reported to have 45% of their genomes attributed to TEs (Duplessis et al. 2011).

Eukaryotic TEs are divided into two classes, depending on their mode of transposition: Class I elements (retrotransposons), which mobilize by a ‘copy-and-paste’ mechanism via a RNA intermediate, and class II elements (DNA transposons), which move by a ‘cut-and-paste’ mechanism via a DNA intermediate (Casacuberta and Santiago 2003; Feschotte and Pritham 2007).

The retrotransposons (class I elements) are the most common TE in fungi (Muszewska et al. 2011). This was found to be also the case in myrtle rust with the frequency of retrotransposons more than five times higher than DNA transposons (Table 3). Retrotransposons can be classified into two types – LTR retrotransposons and non-LTR retrotransposons (encompassing LINEs and SINEs elements), depending on whether they possess or lack long terminal repeats (LTRs) at both ends. The two main superfamilies of LTR retrotransposons found in fungi are *Gypsy* and *Copia*, which differ in the order of RT, ribonuclease H (RH), and IN domains in the virus-like polyprotein (POL; *Gypsy:* PR-RT-RH-INT, *Copia:* PR-INT-RT-RH). These two families comprise the largest proportion (−22%) of TEs in the myrtle rust genome (Table 3). The SINEs do not encode a functional RT and rely on other TEs for transposition and were not accounted for in this analysis.

The DNA transposons (class II elements) have terminal inverted repeats (TIRs) or a rolling circle replicon mechanism. They contain a ‘DDE motif’, which is the active site of the transposase gene. The transposase gene is flanked by a TIR of variable length and catalyses the ‘cut-and-paste’ process of the DNA transposon. DNA transposons are grouped into superfamilies (e.g. *hAT; Tc1*) based on sequence similarity of the transposase gene.

The superfamilies annotated (Table 3) include Tcl (1.7%), hAT (3.6%) and Mutator (19.3%) and they are signified by the ‘DDE’ motif. This motif is present in 11 of the 19 currently recognized superfamilies of DNA transposons (http://www.girinst.org/repbase/index.html). The other DDE-domain containing DNA transposons (75.4%) remain to be identified.

The non-DDE domain-containing DNA transposons in the myrtle rust genome (potentially eight superfamilies) if present, are also yet to be accounted. These non-DDE domain-containing DNA transposons are probably nonau-tonomous and use transposase encoded by autonomous elements located elsewhere in the genome. Hence the percentage of DNA transposons will be potentially higher than the estimated 5% (Table 3).

The contigs with retrotransposable elements, retrotransposons and DNA transposons have higher than mean coverages of contigs, with very high maximum coverages of >10,000 (see Results and Table 4). The group of hypothetical proteins also has a slightly higher than mean coverage with maximum coverage of >5000 (Table 4), suggesting that some of these hypothetical proteins are amplified or repeated in the genome. The very high maximum coverages of retrotransposons and DNA transposons and the unidentified repeat elements in the group of hypothetical proteins suggest that the percentage of repeat elements in the myrtle rust genome is higher than 27%.

These TEs have caused serious difficulties in sequence assembly. The CLC-Genomics Workbench was unable to build longer contigs using the scaffolding function from the many primary contigs assembled, suggesting the presence of a large proportion of branching nodes due to ambiguities from repeated elements. This explains for the large discrepancy between observed contig sizes and the theoretical expected lengths of contigs (more than 100K bp) based on the Lander-Waterman model (Lander and Waterman 1988) for 46 million reads of length 250 bp from the myrtle rust genome. Further work involving the preparation and sequencing of mate-pair libraries will be necessary to scaffold the large number of contigs into longer ones.

### Phylogenetic analysis

The study of evolutionary relationships in Pucciniaceae had reported the use of *18S rRNA* (Wingfield et al. 2004), *28S rRNA* (Maier et al. 2003), partial ß-tubulin and elongation factor1? gene sequences (Van Der Merwe et al. 2008). The *COXI* gene is used in DNA barcoding in animals (http://www.dnabarcoding101.org/) and has been reported to be valuable for the identification of some fungi, *Penicillium* species (Seifert et al. 2007) and cereal rusts (Liu and Hambleton 2012). The availability of sequences for a short segment of the mitochondrial *COXI* gene for a large number of rust species facilitated the use of the short *COXI* gene segment for an analysis of evolutionary relationships of myrtle rust with other rust species. Results of the phylogenetic analysis using criteria including distance, parsimony and likelihood were in agreement with results from analysis of 18S and 28S rDNA data.

Analyses using partial DNA sequences of *18S* and *28S rRNA* genes (Figure 3, Figure S1) and the *COXI* gene (Figure S2) had resolved the rust species used in this study into two distinct, major clades of Pucciniales, Pucciniaceae and Melampsoraceae in agreement with previous studies (Maier et al. 2003; Wingfield et al. 2004, 2007; Van Der Merwe et al. 2007) and placed myrtle rust in the Pucciniaceae clade. Myrtle rust is on a separate branch and not clustered with representatives of rust species in the families of Pucciniaceae, Phragmidiaceae, Sphaerophragmiaceae, Phragmidiaceae, Uropyxidaceae, Chaconiaceae and Phakopsoraceae (Figure 3, Figures SI and S2). Further work is thus required to determine the family placement of myrtle rust in the Pucciniaceae of Pucciniales.

### Implications and recommendations

This study has found that the myrtle rust genome has about 27% of TEs. They may be major players in the generation of genetic variability in the pathogen's adaptation to the environment and new hosts.

The very wide host range of myrtle rust in so many genera would imply that the myrtle rust pathogen, like the cereal rusts, is potentially a complex with taxonomic levels varying from races to species. Simpson et al. (2006) documented eight taxa of rusts on Myrtaceae with different host ranges and specificity. A review had reported 27 synonyms for *P. psidii* (Glen et al. 2007), all of which were found on Myrtaceae hosts. All this suggests the myrtle rust genome may continuously evolve to overcome host resistance, and this co-evolution with their hosts may lead to the generation of a rust complex with different host-specific genotypes.

Rusts in Pucciniaceae have been documented to co-evolve with their angiospermous hosts to enable them to jump across hosts of different species and genera (Van Der Merwe et al. 2008). *Eucalyptus* rust is apparently a specialized genotype evolved from a rust on Myrtaceae in South America to enable it to ‘host jump’ and infect *Eucalyptus*, an introduced Myrtaceae species to Brazil (Ferreira 1983) and hence its name. Isolates from guava did not infect *Eucalyptus* and vice versa (Ferreira 1983) indicating an occurrence in the evolution of a host-specific genotype.

The ‘plasticity’ of rusts to adapt to new hosts pose a huge threat on commercially important Australian *Eucalyptus* and some endangered Myrtaceae genera in the Australian flora. It is thus very important to have an on-going, long-term programme to understand and manage the pathogen. Critical areas would include the study of the genetic diversity of this pathogen and their associated host range and to monitor the evolutionary changes occurring with respect to changes in pathogenic potential and host specificity.

Further research on genetic diversity studies in myrtle rust should include the closely related guava rust and eucalyptus rust to determine the taxonomic family of this rust group in Pucciniaceae. Accurate diagnosis and identification of the different genotypes in this rust complex is crucial for the development of more efficient management strategies and breeding programmes for resistant germ-plasm. Accurate identification of the myrtle rust isolates is also crucial for the regulation of movement of Myrtaceae materials, particularly *Eucalyptus* between geographical areas and countries.
